# Early features of Kawasaki disease with pyuria in febrile infants younger than 6 months

**DOI:** 10.1186/s12887-018-1362-x

**Published:** 2018-12-20

**Authors:** Seo Hee Yoon, Dong Soo Kim, Jong Gyun Ahn

**Affiliations:** Department of Pediatrics, Severance Children’s Hospital, Yonsei University College of Medicine, 50-1 Yonsei-ro, Seodaemun-gu, Seoul, 03722 South Korea

**Keywords:** Differential diagnosis, Fever, Infant, Kawasaki disease, Urinary tract infection

## Abstract

**Background:**

Children with Kawasaki disease (KD) and pyuria have been misdiagnosed with urinary tract infection (UTI). We compared clinical and laboratory features at admission between two groups of infants under 6 months of age who showed initial pyuria, to identify the initial clues suggestive of KD.

**Methods:**

We retrospectively reviewed the medical records of children with fever who were under 6 months of age with pyuria, over a 10-year period (2007–2017). We included infants with sterile pyuria who were finally diagnosed with KD and those with UTI.

**Results:**

During the period investigated, 12 (9.9%) KD patients with sterile pyuria and 378 infants with UTI were included in this study. Older age (*P* < 0.01), a longer duration of fever; total and before admission (*P* < 0.01), more negative nitrite test (*P* < 0.01), higher platelet count (*P* = 0.04), increased C-reactive protein (CRP) (*P* < 0.01) and erythrocyte sedimentation rate (ESR) (*P* < 0.01), were identified as initial features of infants finally diagnosed with KD. In the receiver operating characteristic analysis, optimal cut-off values of 509 k/μL for platelet count, 60 mg/L for CRP, and 68 mm/H for ESR were selected. Patients with ESR > 68 mm/hr had a ninefold higher odds of KD compared to those with lower ESR levels (odds ratio: 8.963, 95% confidence intervals: 1.936–41.493, *P* = 0.005), whereas CRP and platelet count could not significantly increase in the odds of KD at a cut-off point.

**Conclusion:**

Persistent fever, elevated ESR, and negative urine nitrite test can serve as early clues to suspect KD in febrile infants with pyuria.

## Background

Kawasaki disease (KD) is an acute systemic vasculitis of unknown aetiology that affects infants and young children [1]. KD is characterized by inflammation of all the medium-sized arteries and multisystem involvement including the kidney [2]. Renal symptoms in KD include pyuria, prerenal acute kidney injury, haemolytic uremic syndrome, acute nephritic syndrome, nephrotic syndrome, and renal tubular abnormalities [3]. Among them, pyuria is the most frequent renal feature of KD occurring in 30–80% of the patients [4].

Some KD children with pyuria have been mistakenly attributed to urinary tract infection (UTI) [5, 6]. In particular, as infants under the age of 6 months with KD showed higher rate of incomplete presentation as well as pyuria [2, 7–9] that is a significant laboratory marker of UTI with high occurrence rate in these ages [10], it is a diagnostic challenge to distinguish between UTI and KD with pyuria in infants aged < 6 month. Considering that patients with KD younger than 6 months are also known to have a higher risk of developing coronary complications [11, 12], it is necessary that clinicians, who evaluate febrile infant under 6 months of age with pyuria, do not miss the diagnostic potential of KD.

Therefore, it is crucial to find the initial differences among patients with UTI and KD with pyuria to identify KD in febrile infants with pyuria during the early phase of the disease. We studied the clinical and laboratory parameters of infants under 6 months of age who showed fever and pyuria at admission, in order to find initial clues for the early detection of KD that would allow timely initiation of therapy.

## Methods

We retrospectively reviewed medical records of patients under 180 days of age with fever and pyuria who were admitted to Severance Children’s Hospital between January 2007 and January 2017. Among these patients, we included infants who were finally diagnosed as KD or UTI. Diagnosis of KD was based on American Heart Association guidelines [13]. We also included incomplete KD, which showed prolonged unexplained fever, fewer than four of the major clinical manifestations, and compatible laboratory or echocardiographic findings [2]. The definition of pyuria was based on urinary white blood cell (WBC) count > 5 cells/high power field (HPF) and sterile pyuria is defined as pyuria with a negative urine culture [14, 15]. The diagnosis of UTI was based on the culture of a single organism from a catheterized urine culture with a colony count greater than 10,000 or from a clean-catch midstream urine culture with greater than 100,000 colonies [15, 16]. Two or more bacterial species isolated by culture were excluded. We only included patients with KD and with sterile pyuria in the analysis. This study was approved by the Institutional Review Board of our institution (Severance Hospital, IRB number: 4–2017-1199).

### Statistics

Demographic and clinical data are presented as median (range) or frequency. Initial laboratory test data at admission were analysed. Categorical variables were compared using chi-squared test or Fisher’s exact test, and continuous variables were compared by Mann-Whitney test. To compare the predictive capacity of various laboratory tests for diagnosis of KD, receiver operating characteristic (ROC) curves were analysed by estimating the area under the curve (AUC). Optimal cut-off values in the diagnosis of KD were determined according to Youden index [17]. Patients were stratified into two groups according to optimal cut-off values for analyses. Logistic regression analyses were conducted using these groups (higher vs. lower than cut-off value) to estimate odds ratios and 95% confidence intervals. Statistical analyses were performed using SPSS version 23.0 for Windows (SPSS Inc., Chicago, IL, USA) and MedCalc Statistical Software version 18.6 (MedCalc Software, Ostend, Belgium). *P* < 0.05 was considered statistically significant.

## Results

A total of 121 patients under 6 months of age were diagnosed with KD over the study period. Forty-one (33.9%) of the 121 KD children had pyuria. Of the 41 pyuria patients, 12 had sterile pyuria, 7 had concomitant UTI, and 16 had combined bacterial growth which had to be excluded. The other 6 patients were unidentified because they did not perform the urine culture tests. The clinical presentations of the 12 KD infants with sterile pyuria are summarized in Table 1. Two patients satisfied the diagnostic criteria of complete Kawasaki disease, whereas the others were diagnosed with incomplete Kawasaki disease.

**Table 1:** Clinical characteristics of patients with Kawasaki disease with sterile pyuria

Variables	*n* (%) or median (range)
Typical KD/Atypical KD	2/10 (16.7%/83.3%)
Female/male	7/5 (36.1%/63.9%)
Age (months)	5.2 (2.5–5.9)
Total duration of fever (days)	5.5 (3–13)
Duration of fever before admission (days)	4.5 (2–9)
IVIG usage (times)	
1	11 (91.7%)
≥2	1 (8.3%)
Presence of coronary artery lesion (CAL)^a^	3 (25%)
Bilateral coronary ectasia	1 (8.3%)
Left main artery dilatation	1 (8.3%)
LAD dilatation	1 (8.3%)

*KD* Kawasaki disease, *IVIG* intravenous immunoglobulin, *LAD* left anterior descending coronary artery, Data are presented as case number, percentages or median (range)

^a^CAL is defined according to the Japanese Ministry of Health and Welfare guidelines

During the same period, 378 infants under 6 months of age were diagnosed with UTI. Clinical and laboratory findings between KD infants with sterile pyuria and UTI infants were compared (Table 2). Median age of KD group (5.2 months; range, 2.5–5.9 months) was older than that of UTI group (median 3.6 months; range, 0.9–6 months) (*P* = 0.002). Male-to-female ratios in KD and UTI infants were 1.4 (7/5) and 2.8 (277/101), respectively.

**Table 2:** Comparison between patients with Kawasaki disease and patients with urinary tract infection at admission

Parameter (Median, Range)	KD with Sterile Pyuria (*n* = 12)	UTI (*n* = 378)	*P-*value
Age (months)	5.2 (2.5–5.9)	3.6 (0.9–6)	.002
Male/Female	7/5	277/101	.321
Duration of fever (days)			
Before admission	4.5 (2–9)	2 (1–8)	.000
Total duration	5.5 (3–13)	2 (1–16)	.000
WBC (/μL)	17,620 (7180–26,820)	15,130 (3390–33,480)	.232
ANC (/μL)	9390 (4010–15,490)	8035 (100–25,150)	.159
Hb (g/dL)	10.8 (9.5–11.7)	10.9 (8.0–13.3)	.293
Platelet count (k/μL)	532.5 (315–772)	442.0 (162–1492)	.040
CRP (mg/L)	74.6 (31.6–294.5)	33.7 (0.3–191.9)	.001
ESR(mm/hr)	80.0 (27–101)	31.0 (2–120)	.000
Na (mmol/L)	137 (135–141)	138 (114–142)	.352
AST (IU/L)	30 (18–178)	28 (13–507)	.642
ALT (IU/L)	21 (7–53)	22 (7–460)	.658
Albumin (g/dL)	3.9 (3.2–4.7)	3.9 (3.0–4.9)	.903
Total bilirubin (mg/dL)	0.3 (0.3–1.5)	0.4 (0.1–7.1)	.546
Urine β2-MG (mg/L)	0.2 (0.14–0.98)	0.2 (0.01–40.06)	.484
Urine nitrite test (+)	0 (0%)	201 (53.2%)	.000

*KD* Kawasaki disease, *UTI* urinary tract infection, *WBC* white blood cell, *Hb* haemoglobin, *ANC* absolute neutrophil count, *CRP* C-reactive protein, *ESR* erythrocyte sedimentation rate, *Na* sodium, *AST* aspartate aminotransferase, *ALT* alanine aminotransaminase, β2-MG, β2-microglobulin. Data are presented as case number, percentages or median (range)

KD group showed longer duration of fever (before admission and total) compared to UTI group (*P* < 0.01). KD group also showed a significantly higher platelet count (*P* = 0.04), C-reactive protein (CRP) level (*P* < 0.01), and erythrocyte sedimentation rate (ESR) (*P* < 0.01) than UTI group.

UTI group showed higher presence of positive urine nitrite test (*P* < 0.01). There were no significant differences in white blood cell count, absolute neutrophil count, haemoglobin, aspartate aminotransferase, alanine aminotransaminase, albumin, sodium, total bilirubin, and urine β2-microglobulin (β2-MG) (Table 2).

**Fig. 1:**
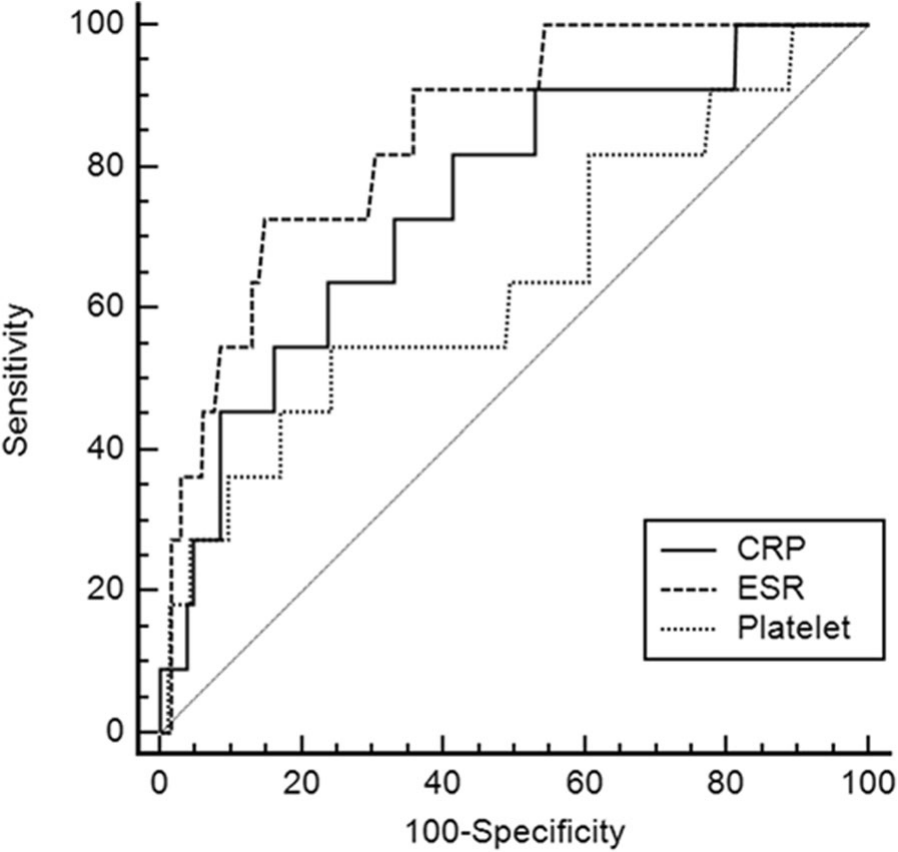
Receiver operating characteristic (ROC) curves for C-reactive protein (CRP), erythrocyte sedimentation rate (ESR) and platelet count for discriminating Kawasaki disease with pyuria (*n* = 12) from urinary tract infection (*n* = 378). The area under the curve (AUC) was 0.674 (95% confidence interval [CI]: 0.625–0.720, *P* = 0.064) for platelet count, 0.749 (95% CI: 0.703–0.791, *P* < 0.001) for CRP, and 0.846 (95% CI: 0.804–0.882, *P* < 0.001) for ESR

We calculated the optimal cut-off values of platelet count, CRP, and ESR for differentiating KD with pyuria from UTI, by drawing ROC curves (Fig. 1). The cut-off value of platelet count to predict KD with pyuria was 509 k/μL with 58.3% sensitivity, 76.5% specificity, 98.3% negative predictive value, and 7.3% positive predictive value (*P =* 0.064). The cut-off value of CRP was 60 mg/L with 66.7% sensitivity, 76.1% specificity, 98.6% negative predictive value, and 8.2% positive predictive value (*P* < 0.001). The cut-off value of ESR was 68 mm/H with 72.7% sensitivity, 85.3% specificity, 99.0% negative predictive value, and 13.8% positive predictive value (*P* < 0.001). The area under the curve (AUC) was 0.674 (95% confidence interval [CI]: 0.625–0.720, *P* = 0.064) for platelet count, 0.749 (95% CI: 0.703–0.791, *P* < 0.001) for CRP, and 0.846 (95% CI: 0.804–0.882, *P* < 0.001) for ESR. Subjects with ESR > 68 mm/hr had a nine-folds higher odds of KD compared to those with lower ESR level (odds ratio: 8.963, 95% confidence intervals: 1.936–41.493, *P* = 0.005) (Table 3).

**Table 3:** Odds ratios for diagnosis of Kawasaki disease using cut-off levels in febrile infants with pyuria

Variables	OR^*^ (95% CI)	*P-*value
Platelet count (> 509 k μ/L)	2.299 (0.633–8.349)	.206
CRP (> 60 mg/L)	2.169 (0.523–8.998)	.286
ESR (> 68 mm/hr)	8.963 (1.936–41.493)	.005

*OR* odds ratio, *CI* confidence interval, *CRP* C-reactive protein, *ESR* erythrocyte sedimentation rate. Odds ratio, 95% confidence interval and *P* value were calculated by binary logistic regression analysis

*Higher vs. lower than cut off value

## Discussion

This study demonstrates that there are different initial features at admission between KD and UTI groups. Older age, longer duration of fever, higher platelet count, higher level of CRP and ESR, and more urine negative nitrite test were observed in KD group compared to UTI group. Our findings can provide early indicators for the early detection of KD in febrile infants with pyuria.

In the present study, the median age of KD group (5.2 months; range, 2.5–5.9 months) was older than that of UTI group (3.6 months; range, 0.9–6 months) (*P* = 0.002). The age difference could be attributed to the fact that neonatal KD is extremely rare [18]. In a previous study, authors examined patients with KD younger than 6 months and reported that the median age was 5 months (range, 2–6) [12], or a mean age was 4.6 ± 3.5 months (range, 2–6) [7]. This is similar to the age range of the KD group in our results. Alternatively, the pooled prevalence rates of febrile UTI aged under 3 months ranges 7.5% (female) to 20.1% (uncircumcised males) [10]. Therefore, pyuria occurring in a febrile infant less than at least 2 months is more likely to be caused by UTI.

The diagnosis of KD is based on clinical criteria, but it could appear and progress in several days after onset of fever. Wu et al. [6] reported two cases of persistent fever and pyuria which were the initial presentation without signs suggestive of KD, and coronary artery abnormalities were noted in both cases. In our study, KD group showed longer fever duration of total and before admission compared to UTI group. In the infants under aged 6 months, prolonged fever might be the only clinical symptom of KD [2]. Thus, early suspicion of KD should be considered in any infant with prolonged fever and culture-negative pyuria.

The CRP, ESR, and platelet count were also higher in KD group than in UTI group in this study. Elevation of acute phase reactants such as CRP and ESR is characteristic laboratory findings of KD [2]. However, they are general indicators of an acute inflammatory process and also increased in UTI, especially upper UTI [19]. Thrombocytosis, classified as a secondary thrombocytosis, is also seen in the upper UTI as well as in KD [2, 20]. Gofrit et al. [21] reported thrombocytosis in a patient with upper UTI is not a random phenomenon, but a marker of kidney obstruction or perinephric abscess. Moreover, ESR > 68 mm/hr showed statistically significant increase in the odds of KD, whereas CRP and platelet count could not significantly increase in the odds of KD at a cut-off point in our study. Therefore, increased ESR itself could also be a good diagnostic marker for KD. However, the laboratory markers should be interpreted cautiously and used as a supportive method to diagnose patients KD using pyuria, in consideration of nonspecificity.

In urinalysis findings except for pyuria, urine nitrite test is helpful for diagnosis of UTI for its high specificity [22]. In this study, UTI group showed higher presence of positive urine nitrite test than KD group (*P* < 0.01). Among UTI patients, 53.2% had positive nitrite test but none in the KD infant. The findings suggest that urine nitrite test could be a useful marker to exclude KD if it is positive.

Sterile pyuria in KD was thought to be due to urethritis caused by a non-specific vasculitis of the urethra and/or the kidney as a result of mild and sub-clinical renal injuries [4, 23]. Urinary β2-MG is being used as a useful indicator to check the function of the renal-urinary tract. In our study, urinary β2-MG is not statistically different in KD and UTI groups. Choi et al. [24] reported that urine β2-MG was elevated in patients with KD and showed no difference between KD with pyuria and without pyuria groups. This result indicates the damage on renal function in most patients with KD.

A major limitation of our study is the small population size, which may have affected our statistical results. Future analysis, with a large sample size, is needed to verify the results of this study. Despite such shortcomings, our data can still provide information regarding initial indicators of KD in febrile infants with pyuria, which will be useful for clinicians.

## Conclusions

Prolonged fever duration, elevated ESR, and negative urine nitrite test can serve as early clues to suspect KD in febrile infants with pyuria. Therefore, in patients presenting with these features as well as persistent fever despite appropriate antibiotic treatment for UTI, echocardiography is warranted to identify the possible presence of KD.

## Abbreviations

AUC: Area under the curve
CI: Confidence interval
CRP: C-reactive protein
ESR: Erythrocyte sedimentation rate
HPF: High power field
KD: Kawasaki disease
OR: Odds ratio
ROC: Receiver operating characteristic
UTI: Urinary tract infection
WBC: White blood cell
β2-MG: β2-microglobulin
